# Physical exertion does not lead to a change in the time taken to correctly recognize stereoscopic stimuli in changing horizontal disparity levels

**DOI:** 10.3389/fphys.2025.1602503

**Published:** 2025-07-11

**Authors:** Florian Frisch, Wolfgang Mehringer, Matthias Ring, Björn Eskofier, Georg Michelson

**Affiliations:** ^1^ Department of Ophthalmology, Friedrich-Alexander-Universität Erlangen-Nürnberg (FAU), Erlangen, Germany; ^2^ Department of Artificial Intelligence in Biomedical Engineering (AIBE), Institute for Machine Learning and Data Analysis, Friedrich-Alexander-Universität Erlangen-Nürnberg (FAU), Erlangen, Germany

**Keywords:** stereoscopic vision, physical exertion, sport, cognition time, depth perception

## Abstract

**Background and question:**

In elite sports, having good stereoscopic vision is important, especially during significant physical exertion. We investigated whether increased physical exertion leads to changes in the time it takes to correctly recognize stereoscopic stimuli in changing horizontal disparity conditions (latency).

**Study design and research methods:**

Twenty-one male and female test subjects, aged 39.0 ± 18.7 years, completed four rounds, each consisting of three attempts. One training round took place with no physical exertion. Then, three rounds took place with low (HR = 100 beats per minute on average), medium (HR = 120 beats per minute on average), and high (HR = 155 beats per minute on average) levels of exertion. These levels of exertion were achieved by using an exercise bike and increasing the power in watts accordingly and by using a pulse oximeter to monitor heart rate. During each attempt, the test subjects were tested for five stereoscopic disparities (32, 128, 192, 256, and 320 arcsec), each presented 16 times for a total of 80 stimuli. Changes in reaction time, percentage of correct answers, and the “GAIN” parameter (reaction time increase per stereo disparity decrease) were recorded.

**Results:**

The percentage of correct answers does not change with increasing physical exertion, and the reaction time for correctly answering does not increase significantly with increased physical exertion. For example, no significant difference was found in the response time at 32 arcsec between low (803.0 m) and high (745.5 m), p = 0.478.

**Conclusion:**

Physical exertion up to heart rates >140/min does not impair the recognition of stereoscopic stimuli at different disparities.

## Background and Question

Stereoscopic vision enables people to perceive depth differences in their environment, ensuring they can safely navigate spatial depths. Stereoscopic acuity is defined as the smallest distance differential between two objects that leads to depth perception. Due to the different positions of the eyes in the head, the corresponding retinal areas create two similar optical images from different perspectives (Dodgson et al., 2004). In adult humans, the pupil distance is 45–80 mm, with an average of approximately 63 mm. The horizontal offset created by these two images is described as horizontal disparity and is measured as an angle in “arcsec.” The smaller the distance differential that can be recognized, the better the horizontal disparity of depth vision. With smaller horizontal disparity, the visual system requires more time to recognize and respond to the stereoscopic stimulus than with greater horizontal disparity, as demonstrated by the results of our working group (Erbes and Michelson, 2021; Kutzner et al., 2022; Schoemann et al., 2017). Reaction time consists of several intervals: a) stimulus reception by the receptor, b) transfer to the visual cortex, c) central processing, d) efferent transfer of motor impulses, and e) muscle latency up to the motor reaction (Pfeifer, 2015).

Reduced stereoscopic vision increases the overall fall risk (Coleman et al., 2007). However, stereoscopic vision is important not only in everyday life but also in certain sports (Salcman et al., 2021). Football players in the National Football League in “skilled positions” (those in which points are immediately scored or prevented) had shorter visual reaction times than those in “non-skilled positions” (e.g., defensive linemen) (OVP, 2024). Basketball players showed a significant correlation between dynamic visual acuity and the accuracy of their shots (Beals et al., 1971). Without binocular visual acuity, the accuracy of their free throws decreased significantly (Vera et al., 2020).

It is not only elite sports, but also modern living and working conditions, that are increasingly characterized by simultaneously performing movement and sensory processing, mostly visual. Many professions require moving and walking while processing information simultaneously (Reiser et al., 2022).

Schmitt et al. proposed an improvement in stereoscopic vision using perceptual learning based on a two-alternative test (recognition of vertical bars in front of or behind a frame of reference) with a varying adaptive critical stereoscopic angle (Schmitt et al., 2002).

In a recently published study, our research group demonstrated that perceptual learning-based vision training using the C-Digital Vision Trainer^®^ significantly improves stereoscopic vision in healthy adolescents and seniors (Erbes and Michelson, 2021; Schoemann et al., 2017). Additionally, digital vision training significantly improved stereoscopic vision in tennis players in terms of critical stereoscopic angle, reaction time, and accuracy (Kutzner et al., 2022).

Zwieko et al. found that physical exertion partially reduced oculomotor efficiency in athletes in terms of binocular vision (Zwierko et al., 2018; Zwierko et al., 2019). A high cognitive burden also appears to negatively affect subsequent physical performance (Brown et al., 2020). However, Reiser et al. proved that increased use of motor skills—in this case, walking on a treadmill—did not always negatively affect processing and performance; often, the effect was positive (Reiser et al., 2022). These promising studies give us the first indications of possible “improvements or deteriorations in stereoscopic reaction time during physical exertion”. Our exploratory cross-sectional study will help to better understand this under-researched area and to develop further scientific research questions, hypotheses and theories.

We asked ourselves if increased physical exertion would lead to changes in the time it takes to correctly recognize stereoscopic stimuli with changing levels of horizontal disparity, as well as changes in “GAIN” and the correctness of the answers given.

## Study design and research methods

### Accuity

Initially, the acuity of each eye was measured using the Freiburg Visual Acuity Test (FrACT) (Bach, 2024). The minimum acuity was 0.7 in the worst eye. On average, acuity was greater than 0.9 (SD 0.32) for 90% of test subjects.

### Research methods

**FIGURE 1:**
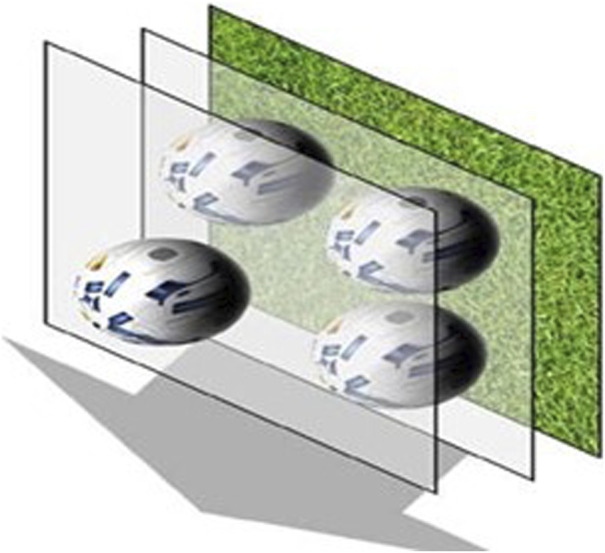
c-Digital Vision Trainer^®^ as a dynamic stereoscopic stimulus test. The targets of the dynamic test, in this case four balls with the same football texture on a grass texture background, constantly move towards the test subject.

The research was carried out using the patented C-Digital Vision Trainer^®^ (Talkingeyes and More GmbH, Medical Valley Center Erlangen, Germany) (Erbes and Michelson, 2021; Kutzner et al., 2022; Schoemann et al., 2017; Paulus et al., 2014). It is a digital visual perception learning system that acts as a dynamic stereoscopic stimulus test presented on a 3D monitor. It provides a moving, stereoscopic stimulus consisting of four balls with a football structure against a grass-textured background (see Figure 1) (Paulus et al., 2014). All four balls move at the same speed toward the viewer. One ball appears closer due to horizontal disparity. This ball emerges from the plane of the other three balls with different horizontal disparity. During movement, the horizontal disparity difference increases until reaching a preset maximum value. However, the viewer always perceives the leading ball as being at the same depth as the other balls (Paulus et al., 2014). The horizontal disparity levels in these areas vary in five preset values: 32, 128, 192, 256, and 320 arcsec. Once the maximum difference is reached, the balls on and in front of the monitor reset to their initial horizontal disparity levels, and the process begins again. In addition to axial movement, each ball uniformly rotates around its x-axis. Test subjects wear 3D glasses, sit on an exercise bike 2 m from the 3D monitor, and use a gamepad to identify the balls approaching them as soon as possible. As soon as an answer is given, the horizontal disparity changes.

### Measurement parameters

Reaction times with the five aforementioned horizontal disparity levels (in arcsec) were measured and recorded, along with whether the answers were correct or incorrect. The system recorded all stereoscopic stimuli with the number 1 for every correctly recognized stimulus and 0 for every unrecognized stimulus, plus the corresponding reaction time. Our study analyzed reaction times for correctly answered questions; that is, the reaction times analyzed came from questions with suprathreshold horizontal disparities.

We also used the “GAIN” parameter, which is the reaction time increase per stereo disparity decrease.

The difference in reaction time between 32 and 128 arcsec and between 128 and 256 arcsec should be considered for each of the three exertion levels. The horizontal disparity value of “32/-128 arcsec” was used due to the longer recognition time, greater error rates, and different error types in the smaller horizontal disparity range (see our results for correctness). These should be compared with the results from the larger horizontal disparity range, “128/-256 arcsec” (lower error rate and shorter recognition time).

Changes in reaction times may depend on improvements in cognition and motor skills. It is assumed that the time portion for purely motor reaction time remains consistent across the five disparities within an individual. To calculate “GAIN,” ignore the motor ability and speed parts; “GAIN” represents a measurement of visual reaction speed (Kutzner et al., 2022).

### Test procedure

The test initially consisted of a training pass without physical exertion (“base measurement”), followed by a short break. This pass was not assessed, and it was repeated three times in a row. Physical exertion was achieved through three additional rounds (3 × 80 stimuli per round) of stereo vision at different levels of exertion on the exercise bike: low, medium, and high.

The “base measurement without physical exertion” allowed the test subjects to become familiar with the system and determine their resting heart rate. Due to fitness, age, nervousness, or general stress, this gave a relatively wide range of 60–90 beats per minute. Due to this large variance, the heart rate values of the “base measurement” served as intraindividual reference points for subsequent exertion rounds. Therefore, for test subjects with a resting heart rate of approximately 60 beats per minute (bpm), the heart rate increased by a factor of 1.25 for “low exertion” (approximately 75–80 bpm), 1.5 for “medium exertion” (approximately 95–100 bpm), and greater than 2 for “high exertion” (greater than 140 bpm). Accordingly, for any test subject with a resting heart rate of 90 beats per minute, there was an increase by a factor of 1.25 to approximately 110 beats per minute for “low exertion.”

During the “low exertion” round, the test subjects’ heart rates were between 90 and 110 beats per minute (100 beats per minute on average). During the “medium exertion” round, the heart rates were between 110 and 130 beats per minute (120 beats per minute on average). During the “high exertion” round, the heart rates were between 140 and 170 beats per minute (155 beats per minute on average), considering the general equation for predicting maximum heart rate (Tanaka et al., 2001).

For each pass, the test subjects were presented with 16 alternate stimuli for each of the five horizontal disparity levels, for a total of 80 possible answers. There were breaks of around two to 5 minutes between individual passes.

The exertion levels were achieved using an exercise bike in a cardiology practice according to national and international guidelines as well as the required knowledge, prerequisites, indications, and contraindications (i.e., stop criteria when pathological abnormalities or stress is observed). Electrical power in watts (W) increased systematically, starting at approximately 50 W, until the required heart rate was achieved (Klingenheben et al., 2018; Sammito et al., 2020).

Oxygen saturation and heart rate were monitored continuously using a finger pulse oximeter from Masimo Corporation (52 Discovery Drive, Irvine, CA 92618, United States).

Each test subject began with a “base measurement without exertion” training round consisting of three passes. The three exertion levels, each with three runs, were carried out alternately according to a randomly selected block randomization of the test sequence, in order to balance the sequenze effect such that every test subject did not start with the same conditions. There were six configurations in which the three exertion levels (“low”, “medium”, and “high”) were carried out. 1) “low-medium-high,” 2) “low-high-medium,” 3) “medium-high-low,” 4) “medium-low-high,” 5) “high-medium-low,” and 6) “high-low-medium.” Configurations 1, 3, and 5 were each selected for four test subjects; configurations 2, 4, and 6 were each selected for three test subjects, in the aforementioned order. There was an approximate 5-min break between each of the three exertion levels until the next higher or lower level was achieved and the passes were started.

### Test subjects

The study included a total of 21 test subjects (16 male and 5 female), who were physically and ophthalmologically healthy and aged 39.0 ± 18.7 years. The test subjects were randomly selected from acquaintances, students, and flyer recipients at the University of Erlangen-Nuremberg. There were no special inclusion or exclusion criteria regarding “healthy test subjects” (including age, history of eye surgery, optic nerve disorders, general diseases, or substance use that affects reaction time). However, an anamnesis was collected before the start of the tests to rule out eye and circulatory diseases. All participating test subjects had no eye or neurological diseases and were non-smokers.

The research was carried out according to the WMA Declaration of Helsinki and information and written consent with reference to the final chapter “Compliance with ethical guidelines”.

### Statistics

The statistics were compared and analyzed for the correctly recognized mean and median values of all 21 test subjects for each of the five horizontal disparity levels and exertion levels. The standard deviation and interquartile range were also calculated. The “GAIN” was calculated and represented graphically, along with the difference in reaction times at low (32/128 arcsec) and high (128/256 arcsec) horizontal disparity levels. The correctness of the results was tested for significance based on the total number of stimuli that were recognized or unrecognized at each horizontal disparity level and exertion level (“medium,” “low,” or “high”). A Poisson regression analysis was performed. The dependent variable was the count variable “sum of correct answers.” Additionally, the percentage for each horizontal disparity level and exertion level was calculated.

Statistical calculations were performed based on missing values (NA), and the data were cleaned or corrected as necessary. The significance level was determined at α = 0.05. Outlier correction was performed. Values that deviated from the mean by more than ±3 were discarded. The hypotheses were tested using ANOVA (analysis of variance) (Eid et al., 2017). This test was performed based on connected measurements or repeated measurements, which are a special form of variance analysis. The interaction was considered significant when the respective p-value was less than 0.05. The Bonferroni *post hoc* test was used to determine which groups presented significant effects. To apply a t-test to two independent samples, the dependent variable, independence of the two samples, normal distribution, and homogeneity and unknown population variance of the samples were required (Shapiro and Wilk, 1965). The Shapiro-Wilk test was used to test for normal distribution (Shapiro and Wilk, 1965), and Levene’s test was used to test for homogeneity of variance (Levene, 1960). According to the central limit theorem, the test size results in a t-distribution if >30 (Ratcliffe, 1968). If the conditions for a two-sample t-test are not met, the Wilcoxon rank sum test or U-test is used instead (Eid et al., 2017). A particular feature of the testing process was how rank ties were handled. If rank ties existed only within a group, the average ranking was assigned (Bortz and Lienert, 2008). If rank ties existed between groups, an exact rank test was performed (Hothorn and Hornik, 2001).

## Results

Does physical exertion lead to altered 3D visual acuity? Based on the three parameters of reaction times, “GAIN,” and correctness, this question should now be answered.

### Reaction times

**FIGURE 2:**
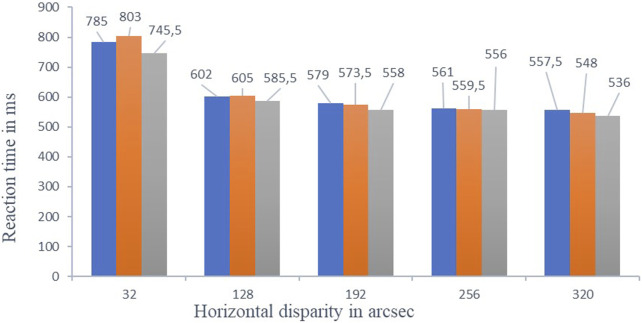
Shows an interindividual overall comparison of the correctly recognized reaction times, as a median for all three exertion levels with respective reaction time in ms. The three grouped columns from left to right represent the exertion level with respective color coding “blue = low exertion, orange = medium exertion and grey = high exertion” in the respective five horizontal disparity levels. The reaction times within the respective horizontal disparity levels show no significant changes.

The median reaction times were close with an increase in exertion by a factor of 1.25 (“low exertion”), 1.5 (“medium exertion”), and greater than 2 (“high exertion”) in relation to the “base measurement without physical exertion” and heart rate at rest. See Figure 2.

The reaction times of all 21 test subjects decreases as disparity increases (32–320 arcsec) in terms of the median (IQR = Interquartile range) with “low 785.0 ms (420.0 ms) – 557.5 ms (211.5 ms), medium 803.0 ms (402.5 ms) – 548,0 ms (227.5 ms) and high exertion” 745.5 ms (357.5 ms) – 536 ms (243.0 ms).

The most noticeable improvements within a horizontal disparity level were 32 arcsec between “medium exertion” (803 m) and “high exertion” (745.5 ms). This gave an improvement of 57.5 ms, so 7.2%. The hypotheses are tested using analysis of variance for data with repeated measures. In the test performed F (3, 60) = 1.125, p = 0.478 not significant (η^2^ = 0.036).

No significant changes in reaction time were found between conditions (“low,” “medium,” and “high exertion”) at a given horizontal disparity level. This indicates that physical exertion did not significantly improve the ability to recognize stereoscopic stimuli.

**FIGURE 3:**
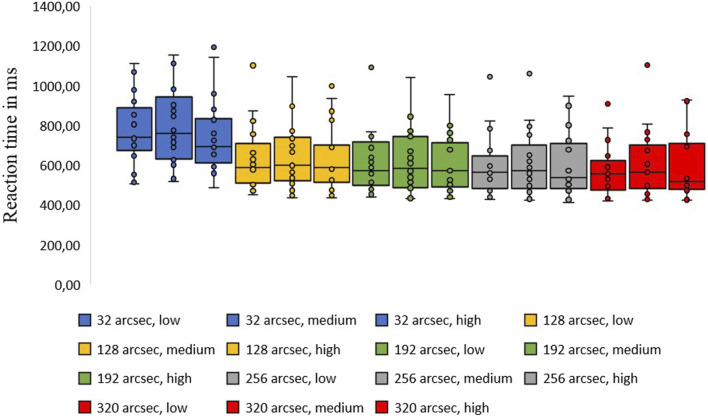
Presents an intraindividual comparison of the 21 median values of all test subjects based on box plots with the “lower quartile Q1 and the upper quartile Q3”, “whiskers” and “outliers”. The five color codes represent the different levels of horizontal disparity as described in the legend, and each box per level from left to right per exertion level of “low, medium and high”. The spread of reaction times and the non-significance between the stress levels “low, medium and high” can be clearly seen.

Figure 3 shows an intraindividual comparison of all 21 test subjects, illustrating the variance in reaction times and the lack of significance between the three exertion levels.

Reaction times decreased as disparity increased within the “low, medium, and high” exertion levels used. At low horizontal disparity levels (32 and 128 arcsec), these decreases were significant: “low exertion,” p = .004; “medium exertion,” p = .001; and “high exertion,” p = .008. The hypotheses were again tested using analysis of variance for data with repeated measures. In the test performed, F (3, 60) = 14.566, p < 0.001 significant (η^2^ = 0.3).

Compared to higher horizontal disparity levels (128 and 256 arcsec), the values for “low exertion” (p = 0.567), “medium exertion” (p = 0.447), and “high exertion” (p = 0.486) were not significant. This means that the smaller the horizontal disparity difference, the longer the recognition process. Below are the results from our research groups (Erbes and Michelson, 2021; Kutzner et al., 2022; Schoemann et al., 2017).

### GAIN = increase in reaction time/decrease in horizontal disparity in ms/arcsec

The following presents the time intervals required by the visual system to process the decrease in stereoscopic difficulty from 32 to 128 arcsec and from 128 to 256 arcsec. As stated in the section “Measurement Parameters,” this research will be limited to these two increases in difficulty and tested for significance using ANOVA (analysis of variance) for data with repeated measurements.

**FIGURE 4:**
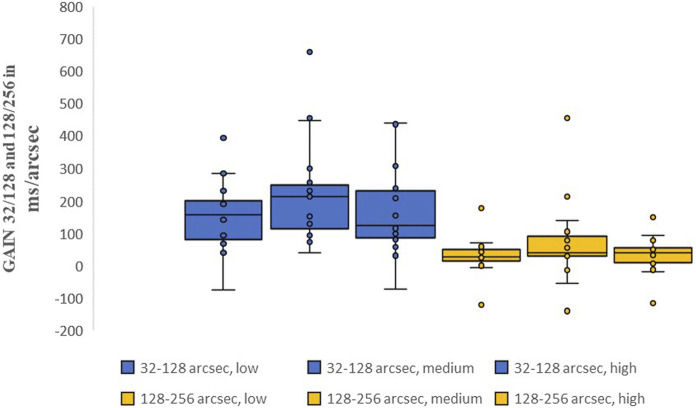
Median intraindividual reaction time change per disparity decrease (“GAIN 32–128”and “GAIN 128–256 in ms/arcsec”) for all 21 test subjects based on box plots with the “lower quartile Q1 and upper quartile Q3”, “whiskers” and “outliers”. The two color codes blue and yellow represent the two horizontal disparity levels used “32/-128 and 128–256 arcsec”, with each value from left to right representing “low, medium and high” exertion. There are also no significant changes in the intra-individual reaction time changes per decrease in disparity with an increase in physical exertion.

Figure 4 shows the change in reaction time for each exertion level and horizontal disparity value. For the three exertion levels (“low,” “medium,” and “high”), the interindividual “GAIN 32/128″ms/arcsec values were 183 m/arcsec (“low”), 198 m/arcsec (“medium”), and 160 m/arcsec (“high”). There was a slight deterioration of 7.6% between “low-medium” (p = 1). For “medium-high,” the values improved by 19.2% (p = 0.587). For “low-high,” the values improved by 12.6% (p = 1). In the test performed, p = 0.587 not significant (η^2^ = 0.001).

The median “GAIN” value was 128/256 m/arcsec for low, medium, and high exertion, which equaled 41, 45.5, and 29.5 m/arcsec, respectively. The greatest improvement, 35%, occurred between “medium” and “high” exertion levels. The other “GAIN” values (32/320, 256/320, and 192/256 m/arcsec) were also tested statistically for the three exertion levels (“low,” medium,” and “high”), but no significant changes were found.

### Correctness

With “low exertion,” 2,149 stimuli (absolute frequency) or 53.7% (relative frequency) of the stereoscopic stimuli were recognized correctly after statistical outlier correction at 32 arcsec, and 1,851 stimuli (absolute frequency) or 46.3% (relative frequency) ±210.7 were recognized incorrectly. With “high exertion,” the relative frequency was also 53% (p = 1). This value increased significantly with an increase in disparity from 32 arcsec to 128 arcsec, reaching 89% (p < 0.001) for “low exertion” and 88% for “high exertion.” For other disparities (192–320 arcsec), values varied between 87% and 91%, regardless of exertion level.

The percentage of correct answers did not change as physical exertion increased. Significance was only found for the “sum of correct answers” when stereo disparity increased from 32 to 128 arcsec (similar to reaction times), but exertion levels had no influence on this.

## Discussion

In conclusion, physical exertion does not significantly affect correctly recognized reaction times, increases in reaction time with decreases in horizontal disparity, or the correct identification of stereoscopic stimuli.

As previously mentioned, a high degree of stereo vision is important and required, especially in elite sports. Studies have shown that stereoscopic vision significantly influences performance in certain sports (Salcman et al., 2021). Several studies have proven that sports such as badminton, baseball, pole vaulting, high jumping, and volleyball require quick spatial orientation decisions (Salcman et al., 2021; Jendrusch, 2019; Laby et al., 2011; Presta et al., 2021). However, stereoscopic vision is less relevant in sports involving strategy, such as football (Paulus et al., 2014). If we look at individual intra-individual changes in reaction times (see Figure 3), there are slight improvements with an increase in physical exertion and slight deteriorations in the reaction times of other test subjects, but none that are significant! Thus, only minimal differences between athletes usually determine the results in sport during peak physical performance. This also fits with the findings on stereo acuity in sport mentioned here and with the results of our study. It could be a reason for the non-significance of the stereoscopic reaction time change under physical stress. None of the 21 test subjects received any training on the C-Digital Vision Trainer^®^. There were no prior test runs or similar training. To avoid selection bias, the subjects were randomly selected through the distribution of flyers, as described above. However, the test subjects themselves decided whether to participate in the study. This meant that the group exhibited characteristics or behaviors that distinguished them from non-participants (“self-selection”).

The three “base measurement without physical exertion” passes at the start served as training and helped determine the heart rate at rest. They also helped the test subjects get used to the system and overcome any nervousness. Some individuals initially exhibited slower hand and finger motor skills, which improved with the increasing number of passes. After the “base measurement without exertion” round, three rounds of “low, medium, high” physical exertion began in the configurations described above. The main focus of this research is to create different conditions for the test subjects.

No intraindividual threshold was determined; for example, no minimum detected horizontal disparity difference was established for each test subject as a starting point for all further horizontal disparity levels. Instead, the levels were predefined at 32, 128, 192, 256, and 320 arcseconds to ensure good comparability between subjects. The aim of this study was not to determine a threshold value, however, but rather to determine the detection time (latency) for suprathreshold horizontal disparities with increasing physical exertion. Through suprathreshold presentation, we investigated the recognition times (latency) of the 3D stimuli shown, which are exclusively influenced by cortical structures.

In a series of studies from our research group, we examined reaction times and accuracy in various groups of healthy subjects and individuals with neurological impairments, and we obtained consistent results.

1. There were differences in reaction time and answer accuracy between different suprathreshold horizontal disparity levels. The smaller the horizontal disparity, the longer the recognition time (latency) and the greater the error rate (correctness) (Erbes and Michelson, 2021; Kutzner et al., 2022; Schoemann et al., 2017).
2. Patients with neurological diseases, such as concussions, and post-Covid patients, presented significantly increased reaction times and reduced correctness rates (Mehringer et al., 2023; Kara et al., 2020). The observed impairment in reaction time and accuracy in post-Covid patients could be related to (1) neuronal perception time, (2) motor reactions and high speed (including the eye muscles), or (3) neuronal processing time. These factors may contribute to an increase in reaction time, either individually or in combination.

Yue et al. assumed that latency time at suprathreshold horizontal disparity levels could characterize neurons in the visual cortex and V2 that respond to horizontal disparity (Yue et al., 2020). In a VEP study, Yue et al. showed that P1 and N1, originating from the posterior cingulate cortex, were statistically correlated with 3D visual fatigue. The results of this study indicate a significant correlation between post-Covid-associated 3D visual fatigue and P1 and N1 amplitudes in the right parietal area. Yue et al. concluded that latency could serve as an indicator for assessing post-Covid-associated 3D visual fatigue. They also suggested that post-Covid 3D visual fatigue is correlated with decreased alertness and capacity to process horizontal disparities.

Our research group’s study (Kara et al., 2020) produced similar results. It can be proposed that latency time for perceiving horizontal disparity characterizes 3D vision.

Skrandies et al. demonstrate that fewer neurons in the visual cortex are involved in processing horizontal disparity compared to V2. Higher visual areas, such as V2, are more heavily involved in stereoscopic processing than the primary visual cortex (Skra and ndies, 2001).

One limitation of our study is the large variance in heart rates (60–90 beats per minute) during the “base measurement,” as described in the “test procedure,” and in maintaining the “exertion levels” while performing the tests. The targeted heart rate increases by factors of 1.25 (“low exertion”), 1.5 (“medium exertion”), and greater than 2 (“high exertion”) were largely achieved. However, a slight decrease in heart rate was observed in a few test subjects for the highest physical exertion level despite best efforts; this generally occurred in the last pass and indicates possible fatigue. This limits the generalizability of the results. Furthermore, as mentioned at the beginning, this study is an exploratory cross-sectional study in which the test subjects were examined in a single session. A lack of cognitive distraction during the study could also limit generalizability.

The precise effects of physical exertion on stereoscopic vision or visual processing remain inconclusive, and further analyses and studies are required. Results from the Leibniz Research Centre for Working Environment and Human Factors at Dortmund University (IfADo) indicate that movement, such as walking, positively impacts the processing of visual information (Reiser et al., 2022). Lunghi et al. found that physical activity, such as cycling with breaks, can improve visual acuity and stereopsis in patients with amblyopia (Lunghi et al., 2019).

## Conclusion

Significant physical exertion with a heart rate exceeding 140 beats per minute does not lead to a deterioration in the time required for correct recognition or affect the recognition of stereoscopic stimuli. These promising results provide a foundation for further research, for example, introducing longitudinal tracking instead of the current cross-sectional design, or recruiting professional athletes as a comparison with “ordinary test subjects.”

Our study’s results suggest that stereoscopic abilities adapt well to physical exertion and that human 3D perception remains constant or improves with significant physical exertion.

## Data Availability

The original contributions presented in the study are included in the article/supplementary material, further inquiries can be directed to the corresponding author.
